# Association between c-type lectin-like receptor 2 and microsatellite instability in colorectal cancer: a cross-sectional study

**DOI:** 10.1186/s12885-022-09834-4

**Published:** 2022-07-28

**Authors:** Xin Zhang, Jia-rui Yuan, Xin Wang, Shuang Fu, Rui-tao Wang, Guang-yu Wang

**Affiliations:** 1 Department of Internal Medicine, Harbin Medical University Cancer Hospital, Harbin Medical University, NO.150 Haping ST, Nangang District, Harbin, Heilongjiang People’s Republic of China 150081; 2 Department of Gastrointestinal Medical Oncology, Harbin Medical University Cancer Hospital, Harbin Medical University, NO.150 Haping ST, Nangang District, Harbin, Heilongjiang People’s Republic of China 150081

**Keywords:** C-type lectin-like receptor 2, platelet activation, microsatellite instability, colorectal cancer

## Abstract

**Background:**

As a transmembrane protein, C-type lectin-like receptor 2 (CLEC-2) is mainly expressed on platelets and released into plasma after platelet activation. Activated platelets participate in the regulation of innate immune cells. Patients with different microsatellite statuses have distinct immune profiles. This study aimed to investigate the association of plasma CLEC-2 levels with microsatellite status among colorectal cancer (CRC) patients.

**Methods:**

A cross-sectional analysis of 430 CRC patients from Harbin Medical University Cancer Hospital was conducted. CLEC-2 levels were measured with fasting venous blood samples drawn from each participant before any treatment. The microsatellite status was evaluated with DNA obtained from fresh frozen tumor tissue samples. The other clinical data were collected and recorded based on the medical system records.

**Results:**

CLEC-2 levels were significantly higher among patients with high microsatellite instability phenotype than the stable microsatellite group, adjusting for other confounding variables.

**Conclusions:**

The increased CLEC-2 is associated with the high microsatellite instability subtype of CRC.

## Introduction

Colorectal cancer (CRC) ranked third in incidence and second in mortality according to the global cancer statistics in 2020 [1]. Environmental and genetic factors (gene mutations, chromosomal abnormalities, and epigenetic changes) are involved in CRC development [2]. Microsatellite instability (MSI) is caused by the impaired DNA mismatch repair system, including high MSI (MSI-H) and low MSI (MSI-L). MSI CRC is characterized by a high density of tumor-infiltrating lymphocytes located in the proximal colon, and a better prognosis than microsatellite stabilization (MSS) tumors, accounting for 15% in CRC without a family history [3, 4]. Tumors can induce platelet activation, and activated platelets could promote tumor progression and metastasis [5]. Mean platelet volume (MPV) is considered an indicator of platelet activation and decreased MPV is a poor prognostic marker for many cancers [6–8]. MPV is significantly reduced in the MSI-H subtype of CRC [9, 10].

C-type lectin-like receptor 2 (CLEC-2) is a type II transmembrane protein. It is mainly expressed on activated platelets and megakaryocytes and released into plasma after platelet activation [11]. Podoplanin, the only established endogenous ligand for CLEC-2, is described on the surface of tumors or lymphatic endothelial cells and stimulates platelet activation through CLEC-2 binding to promote blood-borne tumor metastasis or the separation of blood/lymphatic vessels at the developmental stage [12]. However, few studies evaluated CLEC-2 levels in patients with CRC. This study aimed to determine the plasma levels of CLEC-2 in CRC patients and investigate the associations between CLEC-2 levels and MSI status.

## Methods

### Study population

CRC patients aged 18 years and over were invited to participate in this cross-sectional study in Harbin Medical University Cancer Hospital from January 2018 to December 2018. CRC was identified by histology. Patients were excluded with the following condition: (1) any history of radiotherapy, chemotherapy, or other anti-cancer therapy; (2) infection, hematological disorders, hypertension, and diabetes mellitus; (3) medical treatment with statins and acetylsalicylic acid; (4) insufficient clinical data. The trained research assistant collected data about age, gender, body mass index (BMI), smoking status, and drinking status of all eligible participants with a well-designed questionnaire.

The clinical data were collected and recorded based on the medical system records, including albumin, creatinine, white blood cells (WBC), hemoglobin, platelet count, tumor location, tumor size, histological type, histological grade, venous invasion, perineural invasion, T classification, lymph node metastasis, and distant metastasis. The TNM stage was classified according to the 8th edition of the American Joint Committee on Cancer staging system.

### CLEC-2 measurements

A venous blood sample was drawn from each participant under fasting conditions at primary diagnosis before any treatment. The blood samples were collected by tubes containing sodium citrate and separated by centrifugation at 2500 rpm for 10 minutes, and the plasma was stored at − 80 °C until enzymatic analysis. CLEC-2 levels were measured in the hospital clinical laboratory, using commercially available sandwich ELISA kits (Xinle, Shanghai, China, respectively). Samples were measured as duplicates. The intra- and inter-assay variations were below 8 and 10%, respectively.

### MSI analysis

The MSI was evaluated with DNA obtained from fresh frozen tumor tissue samples, using polymerase chain reaction with primers amplifying the microsatellite markers (BAT25, BAT26, NR-21, NR-24, and NR-27). MSI was defined as ≥3 unstable markers, while MSS was < 3 unstable markers. No sample has only two unstable markers.

### Statistical analysis

The numbers and percentages were reported for presenting categorical variables. The mean ± standard deviation (SD) was used for reporting normal distribution continuous data, and the median (interquartile range) for non-normally distributed data. The Student’s t test was used when comparing variables with normal distribution in two groups, and the Mann-Whitney U test for skewed-distributed data. The χ^2^ test was used to evaluate the differences of categorical variables between groups. Binary logistic regression was used to assess the association between clinicopathological factors with MSI-H status. A receiver operating characteristic (ROC) curve was drawn to determine a threshold value of CLEC-2 for differentiating MSS from MSI-H using MedCalc version 15.0. SPSS Statistics version 25.0 (SPSS Inc., Chicago, IL, USA) was used for statistical analysis, and *p* <  0.05 represents statistical significance.

## Results

A total of 430 eligible CRC patients were included in the final analysis, of which 14.88% were MSI-H patients. Baseline characteristics were compared between patients with MSI-H and MSS. CRC patients with MSI-H had higher BMI, WBC, CLEC-2 levels, younger age and lower hemoglobin levels than patients with MSS (Table 1). MSI-H status was significantly associated with tumor location, tumor size, lymphatic invasion, lymph node metastasis, clinical stage, and histological type (Table 2).

**Table 1:** Clinical and laboratory characteristics of colorectal cancer patients with high microsatellite instability and microsatellite stable status

Variables	MSI-H	MSS	***p*** value
Number	64	366	
Age (years)	56.4 ± 11.8	59.9 ± 9.6	0.029*
Female (n, %)	33 (51.6)	148 (40.4)	0.096
BMI (kg/m^2^)	24.5 ± 3.3	23.2 ± 3.2	0.004**
Current smoker (%)	25 (39.1)	158 (43.2)	0.540
Drinker (n, %)	17 (26.6)	122 (33.3)	0.285
Creatinine (umol/L)	80.9 ± 19.7	81.1 ± 18.4	0.929
WBC (× 10^9^/L)	8.07 ± 2.98	6.92 ± 2.30	0.004**
Hemoglobin (g/L)	124.6 ± 27.3	134.3 ± 22.4	0.009**
Platelet count (×10^9^/L)	286.2 ± 112.6	266.8 ± 82.6	0.191
CLEC-2 (pg/mL)	166.1 ± 41.3	133.5 ± 36.1	< 0.001***

Values are means ± SD, median with interquartile range, or number with percentage

*BMI* body mass index, *WBC* white blood cells, *CLEC-2* c-type lectin-like receptor 2, *MSI-H* high microsatellite instability, *MSS* microsatellite stable

*p <  0.05; **p <  0.01; ***p < 0.001 vs. the values of MSS group

**Table 2:** Clinicopathological features of of colorectal cancer patients according to microsatellite instability status

Variables	Total	MSI-H	MSS	***p*** value
	n (%)	n (%)	n (%)	
Tumor location				< 0.001***
Proximal	150 (34.9)	38 (59.4)	112 (30.6)	
Distal	280 (65.1)	26 (40.6)	254 (69.4)	
Tumor size (cm)				0.011*
< 5.0	282 (65.6)	33 (51.6)	249 (68.0)	
≥ 5.0	148 (34.4)	31 (48.4)	117 (32.0)	
Histological grade				0.254
Well/Moderately differentiated	314 (73.0)	43 (67.2)	271 (74.0)	
Poorly differentiated	116 (27.0)	21 (32.8)	95 (26.0)	
Histological type				0.001**
Non-mucinous	368 (85.6)	46 (71.9)	322 (88.0)	
Mucinous	62 (14.4)	18 (28.1)	44 (12.0)	
Lymphatic invasion				0.030*
Absent	331 (77.0)	56 (87.5)	275 (75.1)	
Present	99 (23.0)	8 (12.5)	91 (24.9)	
Perineural invasion				0.527
Absent	365 (84.9)	56 (87.5)	309 (84.4)	
Present	65 (15.1)	8 (12.5)	57 (15.6)	
T classification				0.811
T1 + T2	63 (14.7)	10 (15.6)	53 (14.5)	
T3 + T4	367 (85.3)	54 (84.4)	313 (85.5)	
Lymph node metastasis				0.010**
Absence	267 (62.1)	49 (76.6)	218 (59.6)	
Presence	163 (37.9)	15 (23.4)	148 (40.4)	
Distant metastasis				0.713
Absence	382 (88.8)	56 (87.5)	326 (89.1)	
Presence	48 (11.2)	8 (12.5)	40 (10.9)	
Stage				0.003**
I-II	258 (60.0)	49 (76.6)	209 (57.1)	
III-IV	172 (40.0)	15 (23.4)	157 (42.9)	

Values are number with percentage. Abbreviation as in Table 1. *p < 0.05; **p < 0.01; ***p < 0.001 vs. the values of MSS group

**Fig. 1:**
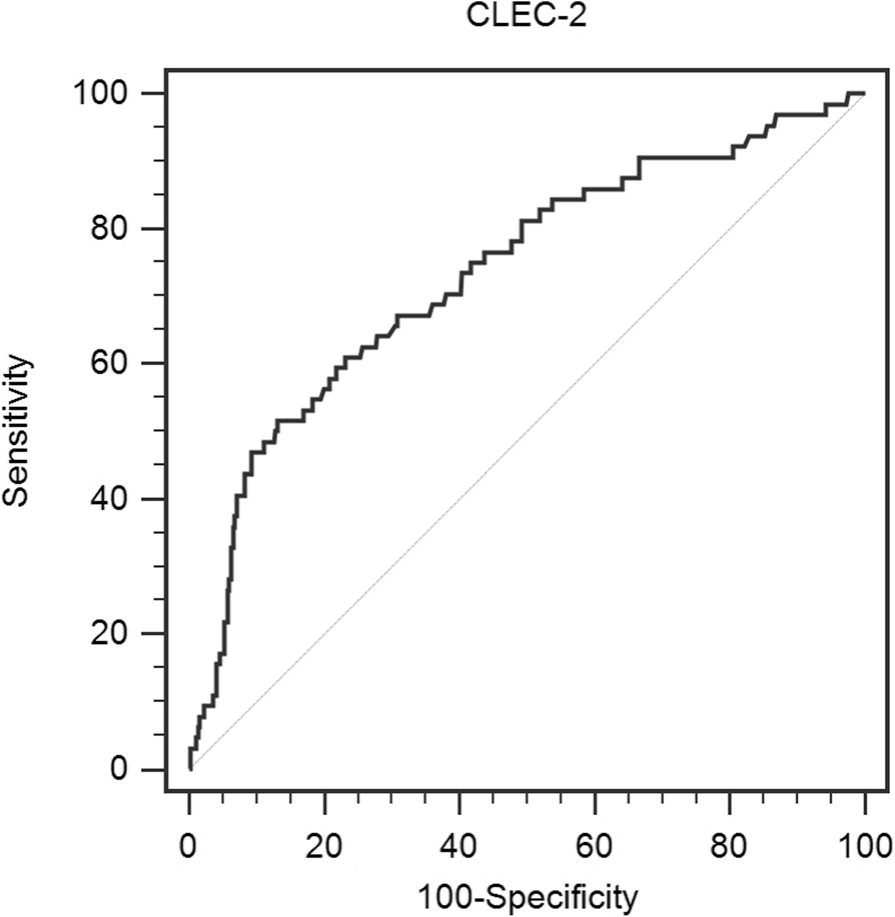
The analysis of receiver-operating characteristic (ROC) curves. Legends: Receiver operating characteristic curve for prediction of high microsatellite instability based on c-type lectin-like receptor 2 (CLEC-2) by enzyme-linked immunosorbent assay

The median value of CLEC-2 was 138.3 (range, 62.0–272.6). The optimal cutoff value determined by ROC analysis for the CLEC-2 was 166.3 for the MSI-H (Fig. 1). The specificity and sensitivity were 51.6 and 86.9%, respectively (AUC = 0.734, 95% CI: 0.663–0.805, *p* <  0.001). CRC patients were divided into two groups according to the cutoff value. Of the 430 patients, 81 patients (18.8%) were detected with CLEC-2 of greater than 166.3 pg/mL, while there were 349 patients (81.2%) whose CLEC-2 levels were less than or equal to 166.3 pg/mL. Association between CLEC-2 and clinicopathologic variables was presented in Table 3. There were significant differences between CLEC-2 levels and MSI status.

**Table 3:** Baseline characteristics of colorectal cancer patients according to c-type lectin-like receptor 2 levels

Variables	Total	CLEC-2 ≤ 166.3	CLEC-2 > 166.3	***p*** value
Age (years)				0.298
≤ 65	309 (71.9)	247 (70.8)	62 (76.5)	
> 65	121 (28.1)	102 (29.2)	19 (23.5)	
Gender				0.432
Male	245 (57.0)	202 (57.9)	43 (53.1)	
Female	185 (43.0)	147 (42.1)	38 (46.9)	
BMI (kg/m^2^)	23.4 ± 3.2	23.9 ± 3.2	23.3 ± 3.2	0.166
Current smoker				0.906
Yes	183 (42.6)	149 (42.0)	34 (42.0)	
No	247 (57.4)	200 (58.0)	47 (58.0)	
Drinker				0.401
Yes	139 (32.3)	116 (33.2)	23 (28.4)	
No	291 (67.7)	233 (66.8)	58 (71.6)	
WBC (×10^9^/L)	7.09 ± 2.44	7.35 ± 3.16	7.03 ± 2.25	0.393
Hemoglobin (g/L)	132.9 ± 23.4	129.4 ± 25.2	133.7 ± 23.0	0.141
Platelet count (×10^9^/L)	270.0 ± 87.8	265.6 ± 82.8	286.9 ± 105.7	0.093
Creatinine (umol/L)	81.1 ± 18.6	83.0 ± 28.4	80.6 ± 15.4	0.467
CEA (ng/ml)	4.37 (2.03–11.31)	3.82 (1.94–13.70)	4.67 (2.12–10.67)	0.768
Tumor size (cm)				0.260
< 5.0	278 (64.7)	230 (65.9)	48 (59.3)	
≥ 5.0	152 (35.3)	119 (34.1)	33 (40.7)	
Tumor location				0.687
Proximal	157 (36.5)	129 (37.0)	28 (34.6)	
Distal	273 (63.5)	220 (63.0)	53 (65.4)	
Histological type				0.383
Non-mucinous	359 (83.5)	294 (84.2)	65 (80.2)	
Mucinous	71 (28.6)	55 (15.8)	16 (19.8)	
Histological grade				0.835
Well/Moderately differentiated	359 (83.5)	292 (83.7)	67 (82.7)	
Poorly differentiated	71 (28.6)	57 (16.3)	14 (17.3)	
Lymphatic invasion				0.285
Absent	331 (77.0)	265 (75.9)	66 (81.5)	
Present	99 (23.0)	84 (24.1)	15 (18.5)	
Perineural invasion				0.545
Absent	365 (84.9)	298 (85.4)	67 (82.7)	
Present	65 (15.1)	51 (14.6)	14 (17.3)	
T classification				0.693
T1 + T2	63 (14.7)	50 (14.3)	13 (16.0)	
T3 + T4	367 (85.3)	299 (85.7)	68 (84.0)	
Lymph node metastasis				0.665
Absence	267 (62.1)	215 (61.6)	52 (64.2)	
Presence	163 (37.9)	134 (38.4)	29 (35.8)	
Distant metastasis				0.707
Absence	382 (88.8)	311 (89.1)	71 (87.7)	
Presence	48 (11.2)	38 (10.9)	10 (12.3)	
Stage				0.392
I-II	258 (60.0)	206 (59.0)	52 (64.2)	
III-I	172 (40.0)	143 (41.0)	29 (35.8)	
MSI status				< 0.001***
MSS	366 (85.1)	318 (91.1)	48 (59.3)	
MSI-H	64 (14.9)	31 (8.9)	33 (40.7)	

Values are means ± SD, median with interquartile range, or number with percentage. MSI, microsatellite instability; Other abbreviation as in Table 1. ***p < 0.001 vs. the values of CLEC-2 > 166.3 group

**Fig. 2:**
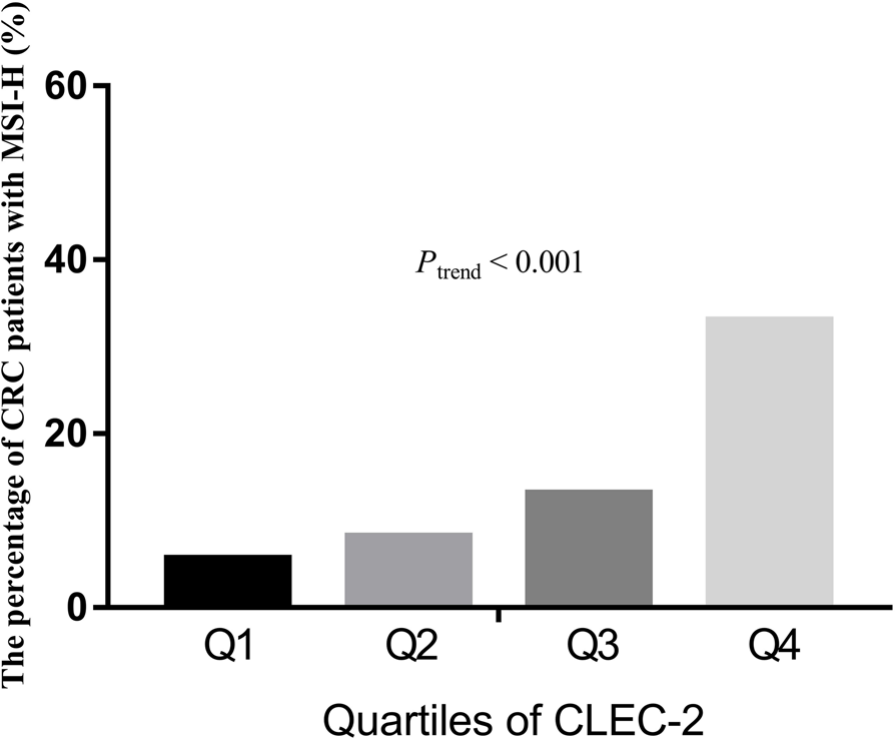
The association between microsatellite instability status and c-type lectin-like receptor 2 levels among colorectal cancer patiens. Legends: The percentage of patients with high microsatellite instability (MSI-H) rose as c-type lectin-like receptor 2 (CLEC-2) levels increased in each quartile among colorectal cancer (CRC) patients

All CRC patients were classified into quartiles according to their CLEC-2 levels, including quartile 1 (Q1) ≤ 104.7 pg/mL, 104.7 pg/mL < quartile 2 (Q2) ≤ 142.7 pg/mL, 142.7 pg/mL < quartile 3 (Q3) ≤ 157.6 pg/mL, and quartile 4 (Q4) > 157.6 pg/mL (Fig. 2). The percentages of CRC patients with MSI-H in each group were 5.6, 8.2, 13.1 and 33.0%, respectively. The results showed that the percentage of patients with MSI-H increased according to CLEC-2 levels.

Logistic regression analysis was performed to evaluate the clinicopathological factors associated with MSI-H status. Eleven variables, including age, BMI, WBC, hemoglobin, CLEC-2, tumor size, tumor location, lymphatic invasion, histological type, lymph node metastasis, and clinical stage, were entered into the original equation. The factors significantly associated with MSI-H in the regression analysis included age, BMI, WBC, CLEC-2, tumor size, tumor location, and histological type (Table 4). Notably, higher CLEC-2 levels were associated with MSI-H after adjusting for other confounding variables.

**Table 4:** Associations between clinical factors and microsatellite instability status

Variables	β	OR (95% CI)	***p*** value
Age (years)	−0.040	0.961 (0.930–0.993)	0.019*
BMI (kg/m^2^)	0.147	1.159 (1.049–1.280)	0.004**
WBC (×10^9^/L)	0.141	1.151 (1.022–1.296)	0.020*
Hemoglobin (g/L)	−0.006	0.994 (0.981–1.007)	0.358
CLEC-2 (pg/mL) (≤ 166.3 vs > 166.3)	2.096	8.132 (4.047–16.341)	< 0.001***
Tumor size (cm) (≥ 5.0 vs < 5.0)	0.949	2.582 (1.332–5.006)	0.005**
Tumor location (Proximal vs Distal)	1.373	3.948 (1.968–7.920)	< 0.001***
Histological type (Mucinous vs Non-mucinous)	1.037	2.820 (1.286–6.187)	0.010*
Lymphatic invasion (Presence vs Absence)	−0.365	0.694 (0.267–1.806)	0.455
Lymph node metastasis (Presence vs Absence)	0.219	1.245 (0.133–11.663)	0.848
Stage (III + IV vs I + II)	−1.031	0.357 (0.039–3.228)	0.359

Values are logistic regression coefficient and odds ratio with 95% confidence interval. β, logistic regression coefficient; *OR* odds ratio, *CI* confidence interval; other abbreviation as in Table 1. *p < 0.05; **p < 0.01; ***p < 0.001 vs. the values of MSS group

## Discussion

The current finding showed that CLEC-2 levels were strongly associated with MSI-H CRC after adjusting the confounding confounders.

As one of the most common cancer, the global burden of CRC is expected to increase by 60% in 2030 [13]. CRC is characterized by the gradual accumulation of malignant transformation involving multiple genetic changes, which is conducive to the proliferation and growth of tumor cells [14]. MMR is an important mechanism used by cells to repair damaged DNA. MMR recognizes and repairs DNA base insertions, deletions, and mismatches, which are caused by DNA polymerase slippage during replication [15]. Compared with normal cells, the mutation rate of cancer cells with MMR is 100 to 1000 times that of normal cells. MSI-H tumors tend to be mucinous and poorly differentiated, with an expansive growth pattern, histological heterogeneity, and tumor-infiltrating lymphocytes [15].

The exact mechanisms of CLEC-2 in MSI-H CRCs are currently unclear. Both CLEC-2 and microsatellite status have been confirmed to be related to platelet activation status, which may be their underlying mechanism [10, 11]. CLEC-2 plays an important role in body development, tumor progression and immune response [16]. In malignant tumors, CLEC-2 and podoplanin work together to activate platelets to promote tumor metastasis and promote tumor-related thrombosis [12]. The difference of CLEC-2 in different CRC subtypes supports the crucial roles that platelets play in immune cells. Previous studies showed that the CD8 T effector gene signature was significantly upregulated in MSI-H tumors compared with MSI-L/MSS tumors [17]. The abundance of podoplanin is related to the immune evasion environment characterized by the infiltration of tumor macrophages and dysfunctional CD8+ T cells [18]. The growth and progression of tumors are also affected by the abundant tumor-associated macrophages in the tumor microenvironment [19]. MSI-H and MSI-L CRC have different tumor microenvironments. MSI-H tumor cells are characterized by their high mutation rate leading to the presentation of mutant peptides on their major histocompatibility complex class I molecules. These processes, in turn, are recognized by immune cells as foreign neoantigens, leading to a high density of cytotoxic CD8+ T cells infiltration and increased levels of IFN-γ secretion. Moreover, CD8 (+) cytotoxic T lymphocytes may increase platelet destruction in immune thrombocytopenia [20].

The tumor cells coated by aggregates of platelets are easy to evade the immune system and adhere to the vessel wall [5]. The activated platelets also secrete various bioactive factors that assist in extravasation to new metastatic sites [21]. Podoplanin has been reported to contribute to cancer pathogenesis by promoting tumor cell invasion and spreading [22]. The increased podoplanin expression was observed in patients with colorectal adenocarcinoma [23]. Clustering of podoplanin by platelet CLEC-2 regulates several molecular pathways involved in tumor cell migration and invasion [24]. Increased plasma CLEC-2 levels were observed in acute coronary syndrome, thrombotic microangiopathy, and acute ischemic stroke, indicating the critical role of CLEC-2 in thrombo-inflammation [25–27].

This study has some potential limitations: Firstly, our results were sourced from a single hospital. Secondly, further study is needed to explain our results mechanistically. Finally, this study only included Chinese participants, and the current finding should be validated in other populations.

## Conclusions

Increased CLEC-2 is associated with the MSI-H subtype of CRC. Further mechanistic research was needed.

## Data Availability

The datasets generated and/or analysed during the current study are not publicly available due to privacy or ethical restrictions in our institute but are available from the corresponding author on reasonable request.

## Abbreviations

CRC: Colorectal cancer
BMI: Body mass index
MVP: Mean platelet volume
MSI: Microsatellite instability
MSI-H: High MSI
MSI-L: Low MSI
MMR: Mismatch repair
MSS: Microsatellite stable
ROC: Receiver operating characteristic
SD: Standard deviation
WBC: White blood cells
